# Quantifying the local strain energy density distribution in the mouse tibia: the critical role of the loading direction

**DOI:** 10.1007/s10237-025-02011-z

**Published:** 2025-09-03

**Authors:** Saira Mary Farage-O’Reilly, Vee San Cheong, Peter Pivonka, Visakan Kadirkamanathan, Enrico Dall’Ara

**Affiliations:** 1 Insigneo Institute, University of Sheffield, Sheffield, UK; 2 Healthy Lifespan Institute, University of Sheffield, Sheffield, UK; 3 School of Medicine and Population Health, University of Sheffield, Sheffield, UK; 4 School of Mechanical, Aerospace and Civil Engineering, University of Sheffield, Sheffield, UK; 5 School of Mechanical, Medical and Process Engineering, Queensland University of Technology, Brisbane, QLD Australia; 6 Centre for Biomedical Technologies, Queensland University of Technology, Brisbane, QLD Australia; 7 School of Electrical and Electronic Engineering, University of Sheffield, Sheffield, UK

**Keywords:** Micro-FE, Micro-CT, Loading direction, Mouse tibia, Mechanical loading, Strain distribution

## Abstract

Understanding how bone adapts to external forces is fundamental for exploring potential biomechanical interventions against skeletal diseases. This can be studied preclinically, combining in vivo experiments in rodents and in silico mechanoregulation models. While the in vivo tibial loading model is widely used to study bone adaptation, the common assumption of purely axial loading may be a simplification. This study quantifies the effect of the loading direction on the strain energy density (SED) distribution in the mouse tibia, a commonly used input for mechanoregulated bone remodelling models. To achieve this, validated micro-finite element (micro-FE) models were used to test the differences in local SED when the bone was loaded along different loading directions. In vivo micro-computed tomography (micro-CT) images were acquired from the tibiae of eleven ovariectomised mice at 18 weeks old before intervention and at 20 weeks old, after six mice underwent external mechanical loading. Micro-CT-based micro-FE models were generated for each tibia at both time points and loaded with a unit load in each Cartesian direction independently. The results from these unit load models were linearly combined to simulate various loading directions, defined by angles *θ* (inferior-superior) and *ϕ* (anterior–posterior). The results revealed a high sensitivity of the mouse tibia to the loading direction across both groups and time points. Several loading directions (e.g., *θ* = 10*°*, *ϕ* = 205–210*°*) resulted in lower medians of the top 5% SED values compared to those obtained for the nominal axial case (*θ* = 0*°*, *ϕ* = 0*°*). Conversely, higher values were observed for other directions (e.g., *θ* = 30*°*, *ϕ* = 35–50*°*). These findings emphasise the importance of considering the loading direction in experimental and computational bone adaptation studies.

**Supplementary Information:**

The online version contains supplementary material available at 10.1007/s10237-025-02011-z.

## Introduction

Bone adapts in response to biomechanical and/or biochemical stimuli (Hadjidakis and Androulakis 2006). For example, trabeculae within a bone form along the direction of stress and in healthy conditions, they can be remodelled if the direction of stress changes (Fyhrie and Carter 1986). The aspect of this phenomenon that relates to the strain within the bone is characterised by Wolff’s law, which states that bone adaptation is triggered by mechanical stimuli (Wolff 1892). Frost’s mechanostat theory expanded upon Wolff’s law by proposing that bone resorption occurs when the bone tissue is strained below a certain threshold, while bone formation occurs when it is strained above another threshold value (Frost 2003). This theory assumes that the osteocytes, embedded in the bone extracellular matrix, sense the mechanical stimuli and orchestrate bone formation by osteoblasts and bone resorption by osteoclasts. As strain contributes to bone adaptation, it becomes possible to utilise mechanical loading in order to strengthen the bone itself (Skerry et al. 1989; Gluhak-Heinrich et al. 2003). Applying these principles, alongside other interventions such as pharmacological treatments, offers significant potential for promoting bone formation to prevent or treat skeletal diseases such as osteoporosis.

Preclinical in vivo animal studies aid in understanding the mechanisms underlying bone adaptation. The ovariectomised mouse model, which induces a skeletal phenotype similar to that observed in postmenopausal osteoporosis, is commonly used to investigate the effects of external mechanical loading on bone adaptation (Bouxsein et al. 2005; Roberts et al. 2019). The mouse tibia loading model is one of the most widely adopted approaches to study mechanically induced bone formation, with at least 58 studies utilising it in the past 15 years (De Souza et al. 2005; Meakin et al. 2014; Nepal et al. 2023; Sugiyama et al. 2012). Additionally, this model has been applied to other murine anatomical sites, such as the ulna and caudal vertebra (Nepal et al. 2023). The majority of these studies investigated the effect of external mechanical loading on the morphometric parameters of the cortical (e.g., cortical thickness) and trabecular (e.g., trabecular bone volume fraction, thickness and number) bone, or the structural mechanical properties (e.g., stiffness and strength) of the mouse tibia (Nepal et al. 2023). However, a detailed analysis of the effect of external loading on the local mechanical properties is missing and could provide deeper insights into predicted mechanoregulated bone adaptation.

In vivo micro-computed tomography (micro-CT) enables longitudinal assessment of bone remodelling by capturing high-resolution images of the same bone over time. It is currently considered the gold standard for measuring bone adaptation in preclinical studies (Bouxsein et al. 2010; Dall’Ara et al. 2016; Van Hoff and Dall’Ara 2019). In vivo micro-CT images of the mouse tibia, captured at subsequent time points, have been used to investigate ageing, drug treatments and mechanical loading on the bone (Birkhold et al. 2016; Buie et al. 2008; Campbell et al. 2014; De Souza et al. 2005; Holguin et al. 2014; Javaheri et al. 2020; Lu et al. 2017). Meanwhile, mechanoregulation computational models, based on Frost’s mechanostat theory, provide an emerging avenue for non-invasive predictions of bone adaptation over time (Carriero et al. 2018; Cheong et al. 2020a, 2020b, 2021a, 2021b; Levchuk et al. 2014; Pereira et al. 2015; Schulte et al. 2013). Some of these mechanoregulation models combine micro-CT-based finite element (FE) models with mechanoregulation algorithms to predict local bone adaptation over time. The FE models predict the distribution of the mechanical stimulus, typically represented by the equivalent strain or the strain energy density (SED) values across the bone, which serve as inputs for the mechanoregulation algorithm that predicts local bone formation or resorption (Carriero et al. 2018; Cheong et al. 2020a, 2021a; Levchuk et al. 2014; Schulte et al. 2013).

Bone adaptation has been predicted in response to physiological loading (Cheong et al. 2020a, 2020b, 2021a, 2021b), external mechanical loading (Carriero et al. 2018; Cheong et al. 2021a; Pereria et al. 2015; Schulte et al. 2013), a combination of a physiological load and external mechanical loading (Cheong 2021a), or a combination of mechanical loading and pharmacological treatment (Cheong et al. 2021b). The mechanoregulation models have varying degrees of accuracy. While they can effectively capture bone formation well (e.g., Cheong et al. (2021b) achieved approximately 80% formation spatial match for the tibia of OVX mice under external mechanical loading, Schulte et al. (2013) achieved a 72% formation spatial match for the caudal vertebra of OVX mice), they are less successful in predicting resorption (Cheong et al. (2021b) captured approximately 35% of resorption sites, Schulte et al. (2013) captured 29%). These findings and the overall model outputs depend on the correctness of the underlying assumptions used to generate the models. For example, Cheong et al. (2021a) showed that local regions of tibial adaptation are affected by the loading conditions. However, only three loading conditions were investigated: a 12N axial load, a physiological load, and a 12N axial load combined with a physiological load. Most of the other models presented in the literature simply assume one loading condition, with a limited understanding of their sensitivity to the magnitude and direction of the external loads that drive the mechanoregulated bone adaptation.

A key assumption commonly made in studies simulating the loading direction experimentally, induced by the in vivo tibial loading model, is that the load is applied axially to the bone. However, as the load is applied experimentally through the knee and ankle joints, the direction of the force applied to the tibia is unknown. Furthermore, as this procedure is repeated several times, repositioning may also have an effect on the local bone strains (Giorgi and Dall’Ara 2018). Recent research using micro-CT-based FE models demonstrates that a change of loading direction within 30 degrees from the axial direction can significantly impact the bone’s structural mechanical properties (Farage-O’Reilly et al. 2024). In fact, the predicted failure load ranged from half to double of that calculated in the assumed axial case. Nevertheless, it is still unknown how the loading direction affects the local mechanical properties in the mouse tibia, which would impact the mechanoregulated bone adaptation.

The aim of this study was to use a validated micro-CT-based FE model to quantify the effect of the loading direction on the SED distribution within the mouse tibia for ovariectomised mice who were either subjected to or were not subjected to controlled external mechanical loading.

## Materials and methods

Data collection and preprocessing were performed in prior studies within the group (Roberts et al. 2019, 2020). Additionally, this study utilises the same validated finite element modelling approach detailed in Farage-O’Reilly et al. (2024). However, the boundary conditions of the models were updated to simulate the nominal load (12N, axial) used in the in vivo tibial loading model (Roberts et al. 2020). The main features of the experiments and of the modelling pipeline are briefly described here.

**Fig. 1:**
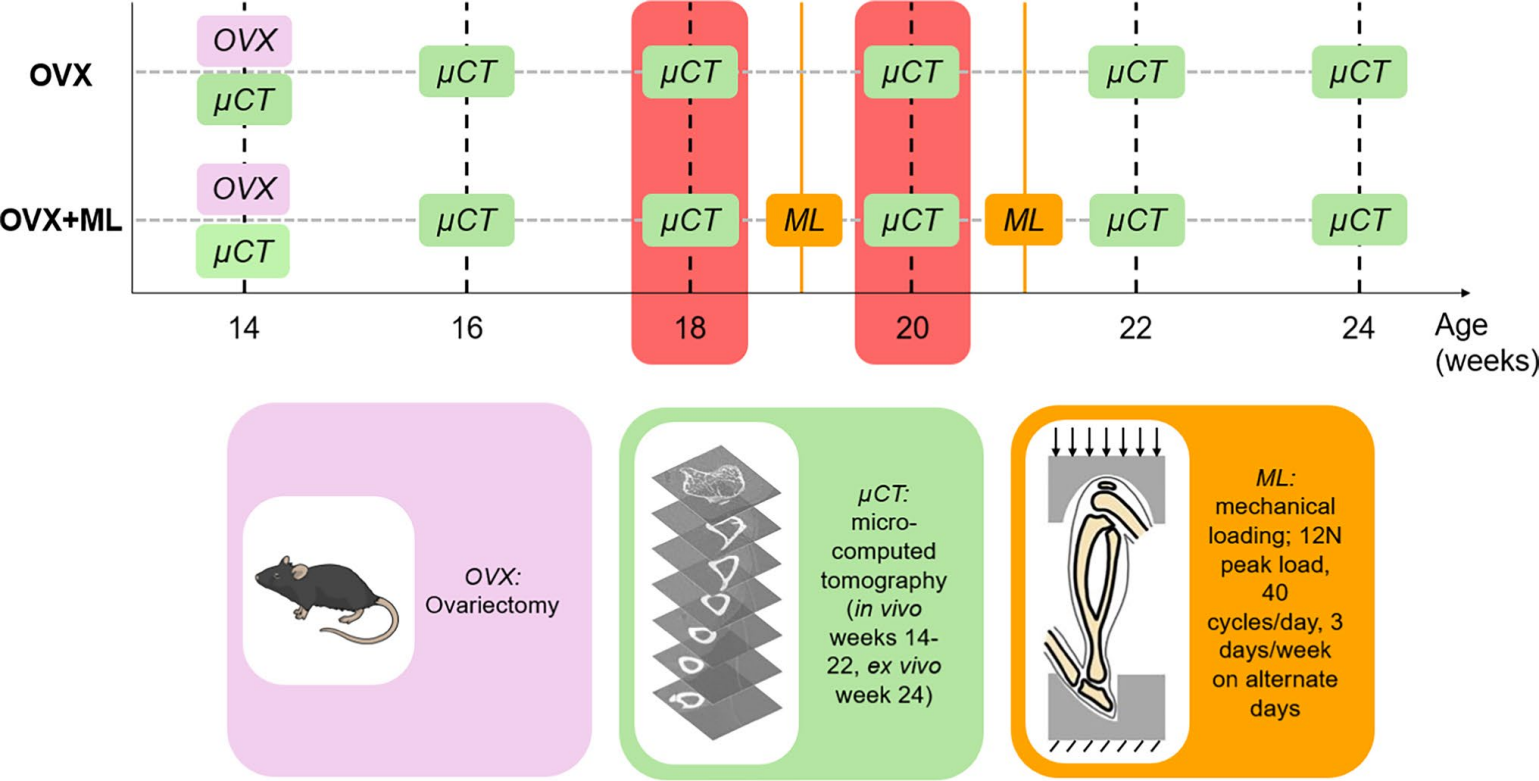
Data collection timeline. Experimental design of the murine experiment performed by Roberts et al. (2020), used in this study. OVX: ovariectomy surgery at week 14 of age, OVX + ML: ovariectomy surgery at week 14 of age followed by controlled external mechanical loading applied at weeks 19 and 21 of age. The whole mouse tibia was micro-CT imaged every 2 weeks. In this study, micro-CT images collected at weeks 18 and 20 of age were used (highlighted in red). Mouse image by NIAID Visual & Medical Arts 2024

Imaging data was acquired from a previous study by (Roberts et al. 2019, 2020) where eleven female C57BL/6 mice were ovariectomised at age 14 weeks (Fig. 1) and the right tibia of each mouse was scanned every other week, from week 14 to 24, using in vivo micro-CT (VivaCT80, Scanco Medical Brütisellen, Switzerland). The micro-CT scanning parameters were defined to scan the whole tibia with a resolution that enables the assessment of the cortical and trabecular bone microstructure with a reasonable radiation dose (Oliviero et al. 2017, 2019): voltage 55*kVp*, intensity 145*µA*, voxel size 10*.*4^3^*µm*^3^, field of view 32* mm*, integration time 100* ms*, samples/projections 1500*/*750. A third-order polynomial beam hardening correction algorithm based on a 1200 mgHA/cm^3^ wedge phantom was used in the reconstruction.

**Fig. 2:**
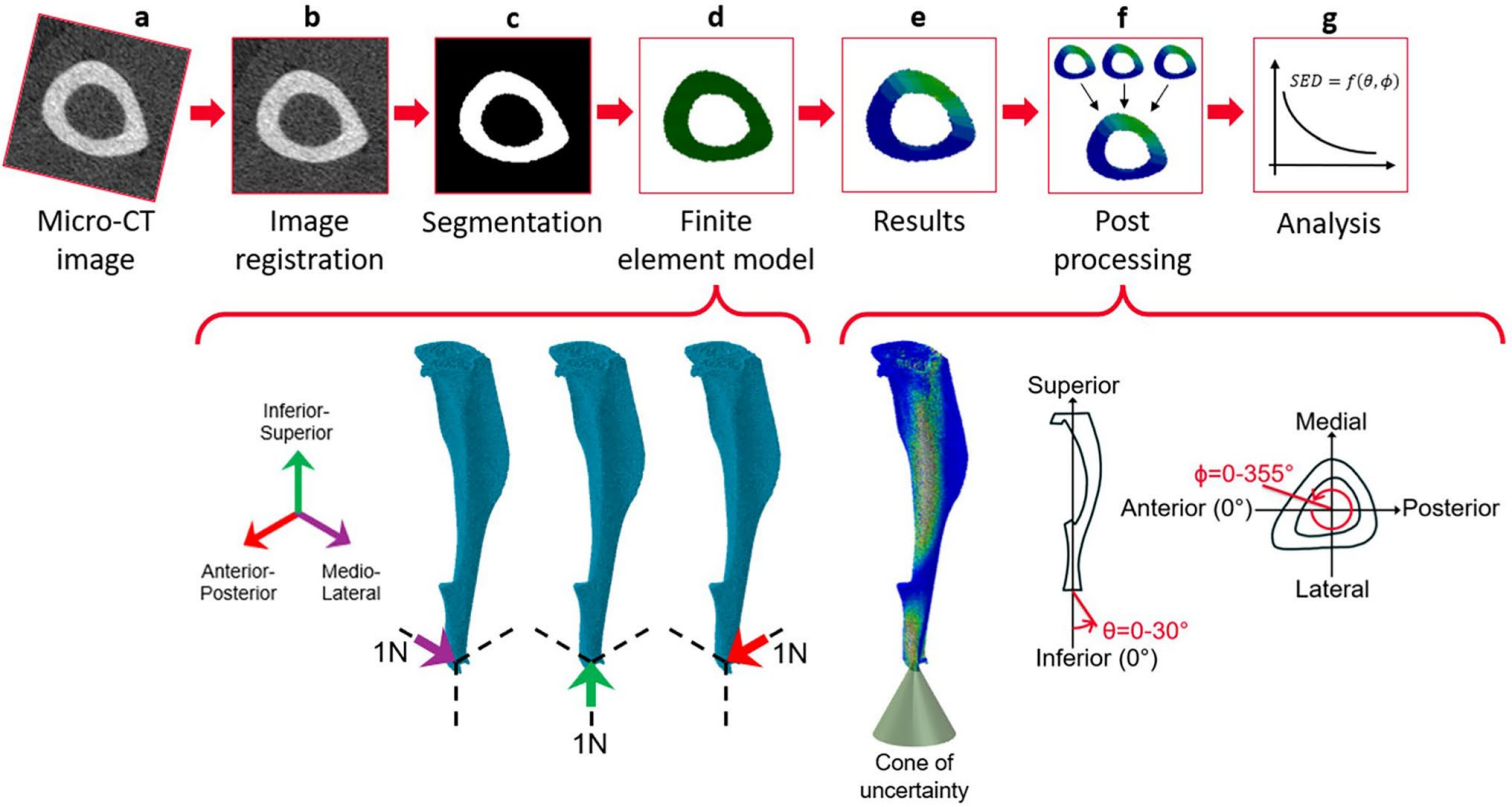
Flowchart illustrating the main steps of the image processing and FE modelling pipeline. **a** Micro-CT imaging. **b** & **c** Image processing. **d** & **e** Finite element modelling – three models with independent unit load cases were solved for each tibia: 1N in the medio-lateral direction, 1N in the inferior-superior direction, or 1N in the anterior–posterior direction. **f** Post-processing was used to calculate the strain energy density (SED) along different loading directions which fall within a cone of possible loading directions, with a resultant force of 12N. The loading directions were defined as a function of two angles θ and ϕ. The angles from the inferior-superior axis (θ) and from the anterior–posterior axis (ϕ) ranged from 0° to 30°, and from 0° to 355°, respectively, both in steps of 5°. **g** The SED distribution across the tibia were analysed and the frequency plots were created

In this study, the micro-CT images acquired at weeks 18 and 20 of age (Fig. 2a) for both the ovariectomised mice (OVX group, N = 5) and for mice ovariectomised and subsequently subjected to controlled external mechanical loading (OVX + ML group, N = 6) were used. To induce mechanoregulated bone apposition, the in vivo tibial loading model was used. The mouse tibia was mechanically loaded in a controlled manner at weeks 19 and 21 (Fig. 1). The loading protocol was defined to apply 40 cycles of load between − 2N and − 12N (compression on the tibia) at a high-strain rate, three times per week on Mondays, Wednesdays and Fridays (ElectroForce BioDynamics 5100, TA instruments, USA). The experimental procedures described in detail in Roberts et al. (2020) were approved by the local Research Ethics Committee of the University of Sheffield and complied with the UK Animals (Scientific Procedures) Act 1986.

In order to exclude potential modelling artefacts induced by the growth plates and the tibio-fibular junction, 80% of the tibia was cropped starting from the first micro-CT slice distal to the proximal growth plate. The fibula was virtually removed (MATLAB, 2018A, The MathWorks Inc., Natick MA, USA) (Cheong et al. 2020b; Lu et al. 2016). While the fibula may affect the distribution of the load and the axial and transverse stiffness of the tibio-fibular structure, modelling it accurately would require the modelling of the proximal tibio-fibular junction and of the soft tissues around the tibia and fibula, which constrain the lateral movements of the bone structures. Considering the lack of reliable material and contact properties for these structures, and that the focus of this study is on the tibia, the fibula was removed before the creation of the micro-FE models. The cropped images at each time point and for each mouse were then rigidly registered (Fig. 2b) using a previously established pipeline. In order to align every micro-CT image of the mouse tibiae (from both time points and from both groups) to the same reference system, the micro-CT image of a mouse tibia acquired at week 14 of age was randomly selected as a reference. This image of the tibia was manually rotated and translated to align its longitudinal axis with the z-axis of the image and so that the sagittal plane bisected the midpoint connecting the centres of the medial and lateral condylar articular surfaces (Lu et al. 2016). The other micro-CT images were rigidly registered to the reference image (Amira 6.3.0, Thermo Fisher Scientific, France), as detailed in Lu et al. (2016, 2017). Reproducibility errors for this procedure were less than 3.5% when estimating the local bone mineral content (BMC) (Lu et al. 2016), 2.0% when estimating the failure load, and 4.1% when estimating the stiffness (Oliviero et al. 2022).

An automatically calculated single-level threshold was used to segment the cropped and registered images (Fig. 2c). The threshold value was identified from the frequency plots of the grey-level image, as the mean value between those associated with the two peaks corresponding to the background voxels and the bone voxels (Cheong et al. 2021b; Oliviero et al. 2018). The segmented bone volume was then converted into a micro-FE model by transforming each bone voxel into a finite element (Fig. 2d). Linear 8-node hexahedral elements were used (Oliviero et al. 2021b). Isotropic, homogeneous, linear elastic material properties (*E* = 14*.*8*GPa*, *ν* = 0*.*3) were assigned to each finite element. Nodes at the proximal end were fully constrained, while those at the distal end were connected via kinematic coupling to a control node positioned at the distal surface centroid with a slight superior offset, to prevent over-constraining of the tibia (Cheong et al. 2020a). The outputs of this micro-CT-based finite element (micro-FE) model have been previously validated against experimental apparent structural measurements of stiffness and strength and experimental local displacement fields measured by using digital volume correlation (Olivero et al. 2018, 2021a, 2021b). In order to calculate the displacements, strains, and SED across the tibia for each loading direction, three independent unit loads along the inferior-superior, medio-lateral, and anterior–posterior directions were applied for each mouse at each time point (Fig. 2). The model outputs were combined during the post-processing step to simulate a 12*N* in magnitude load. The SED was subsequently calculated at each node within the models. Thanks to the linearity of the problem, the principle of superposition of the effects was employed during post-processing (Fig. 2f) to dramatically reduce the required number of micro-FE models. To do so, the SED values at each considered loading direction were calculated by combining linearly the outputs of the three micro-FE models with unit loads. This approach allowed for the assessment of SED distributions for loading along different angles, effectively simulating a cone of loading directions originating from the point of load application. This cone was defined by variations in the inferior-superior axis (*θ*, between 0*°* and 30*°* in 5*°* steps) and rotations around the inferior-superior axis from the anterior–posterior axis (*ϕ*, where 0*°* represents the anterior axis and positive angles are anticlockwise when viewed from inferior to superior, ranging between 0*°* and 355*°* in 5*°* steps) (Fig. 2). In total, 504 loading directions were evaluated for each mouse tibia, including mice from both OVX and OVX + ML groups and from the two time points. An automatic pipeline was developed to create the input files for the micro-FE models (MATLAB, 2018A, The MathWorks Inc., Natick MA, USA), run the models for the unit loads (Fig. 2e, Abaqus 2018, Dassault Systèmes Simulia, RI, USA) on the University of Sheffield High Performance Computing Clusters (ShARC), and postprocess the results (MATLAB, 2018A, The MathWorks Inc., Natick MA, USA).

The frequency plots for the SED nodal values across the tibia were plotted for each loading direction, for 10–90% of the cropped tibia (to minimise any effects due to the boundary conditions). Based on the mechanostat theory, bone formation is anticipated in response to the higher range of SED values experienced by the tibia (Frost 2003; Cheong et al. 2021b). Therefore, the median of the top 5% SED values (95P_SED, *MPa*) was calculated for each mouse for each loading direction to reduce the impact of isolated SED peak values in the distributions, which are likely due to geometrical issues from image artefacts or local stress–strain concentrations. The average of the 95P_SED values was calculated for each group and each time. The mean, standard deviation (SD) and coefficient of variation (CV) of the 95P_SED were calculated for the nominal axial loading direction for each group of mice, at each time point. To enable comparison across loading directions, the 95P_SED for each loading direction was normalised (N95P_SED, %) by the 95P_SED calculated for the nominal axial loading direction (*θ* = 0*°*, *ϕ* = 0*°*) for each mouse at each time point.

The following quantities were calculated for all considered loading directions: The normalised percentage difference of 95P_SED between time points (week 18 vs week 20; ∆95Pt_SED, %), for both OVX and OVX + ML groups, was calculated to evaluate the temporal effects (between week 18 and week 20) on the sensitivity of the SED values for different loading directions; The percentage point (difference between the percentage changes) of the 95P_SED from week 18 to week 20 calculated between the OVX and the OVX + ML groups (∆95Pg_SED, %pt) was calculated to evaluate the effect of mechanical loading on the sensitivity of the SED values for different loading directions.

Considering that the data was not normally distributed (Shapiro-Wilks test) and that the sample size was small, non-parametric tests were used to compare values between different groups. The non-parametric two-tailed Wilcoxon test was used to test differences between the minimum or maximum 95P_SED values across all loading directions between time points within the same group. To test differences between the minimum or maximum 95P_SED values across all loading directions between the groups, the two-tailed Mann–Whitney U test was used. The two-tailed Wilcoxon test was applied to test the effect of the loading direction on the ∆95Pt_SED, while the two-tailed Mann–Whitney U test was used to test its effect on the ∆95Pg_SED. Finally, to test the differences between the nominal axial loading direction and every other loading direction (N95P_SED), the two-tailed Mann–Whitney U test was used. For all statistical analyses, the statistical significance level was set at *α* = 0*.*05.

## Results

**Fig. 3:**
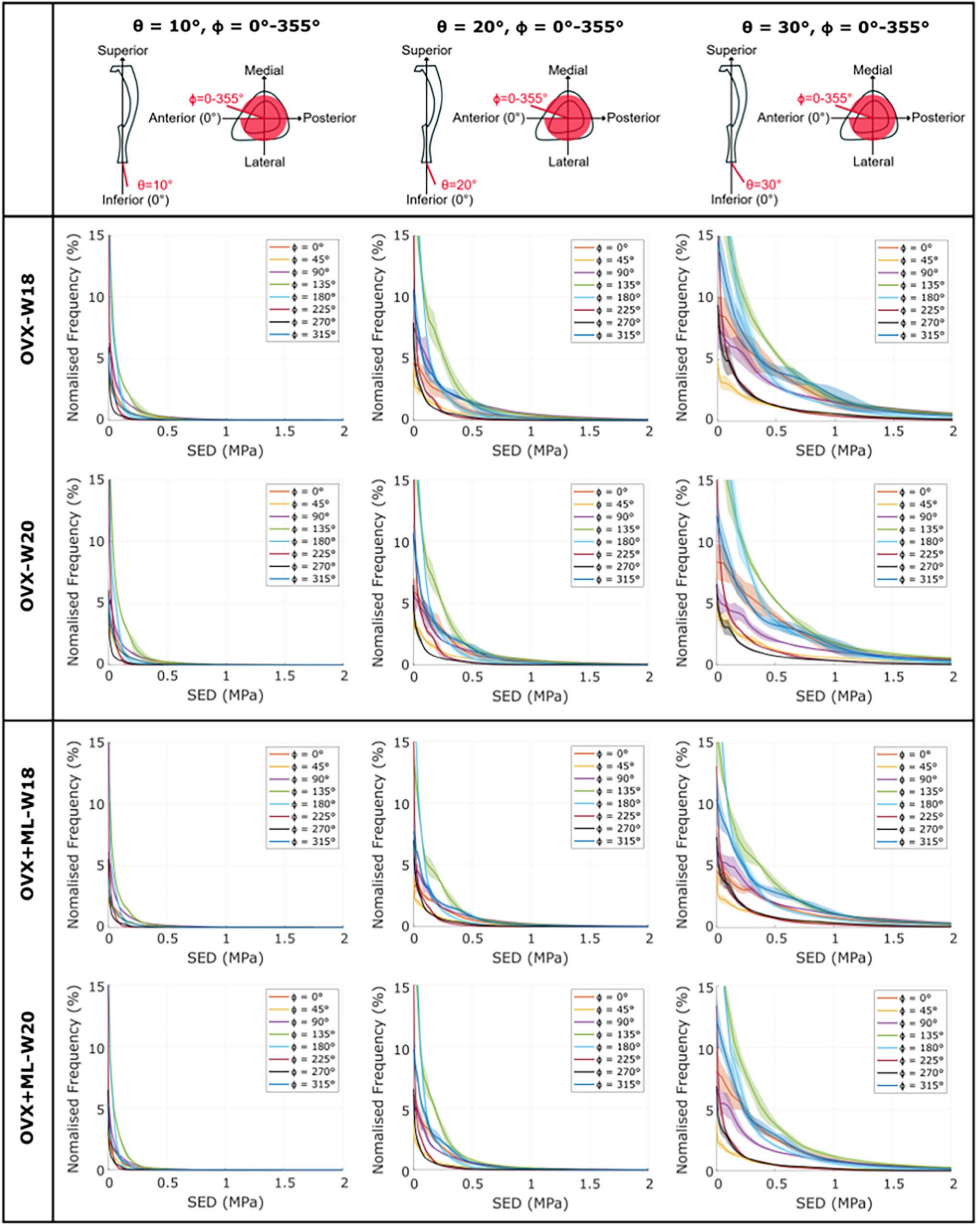
The strain energy density (SED) consistently increased as θ increased for all values of ϕ. The frequency plots for the SED (mean ± standard deviation), between 0 MPa and 2 MPa, across 10–90% of the cropped tibial length, for the loading directions associated with θ = 10°, 20°, 30° and ϕ = 0 − 355°, for both groups at both time points. OVX, ovariectomy; OVX + ML, ovariectomy and mechanical loading; W18, week 18; W20, week 20

**Fig. 4:**
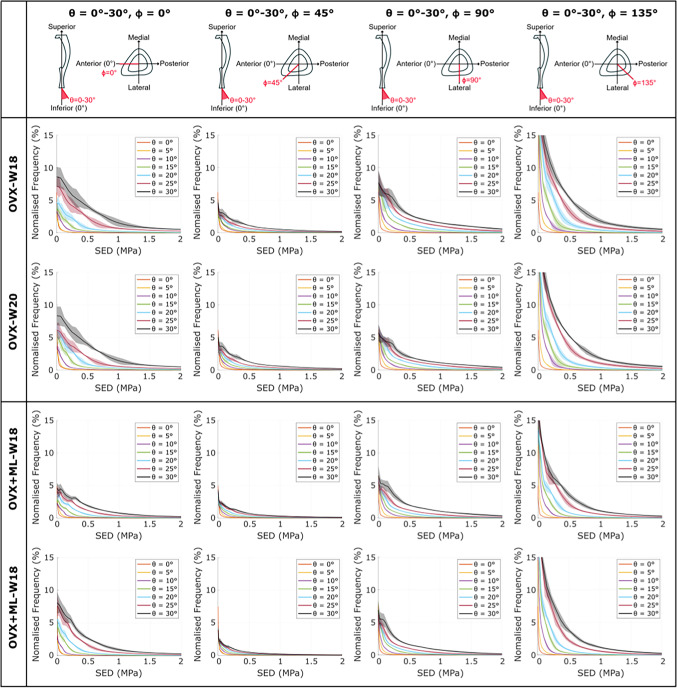
The strain energy density (SED) distribution fluctuated as ϕ increased. The frequency plots for the SED (mean ± standard deviation) between 0 MPa and 2 MPa, across 10–90% of the cropped tibial length, for the loading directions associated with θ = 0 − 30° and ϕ = 0°, 45°, 90°, 135°, for both groups at both time points. OVX, ovariectomy; OVX + ML, ovariectomy and mechanical loading; W18, week 18; W20, week 20

When comparing between loading directions, large variations in the SED distributions across the mouse tibia were observed. As *θ* increased (i.e., as the angle from the inferior-superior axis increased), the SED distribution deviated from the nominal axial case: the SED consistently increased, for most tested values of *ϕ* (Figs. 3 & 4). This was a consistent trend across all groups and timepoints. However, the change in SED distribution fluctuated as *ϕ* increased (Fig. 3). This fluctuation was dependent on the specific loading direction, with the lowest SED fluctuation observed at loading directions associated with *ϕ* = 45*°* and the largest SED fluctuation observed at loading directions associated with *ϕ* = 135*°*. The SED distribution for the loading directions applied in the anterior–posterior (*ϕ* = 0*°*) and medio-lateral (*ϕ* = 90*°*) directions were similar and were distributed between the range of those applied at *ϕ* = 45*°* and *ϕ* = 135*°*. This pattern was consistent across groups and time points.

The largest changes in the SED distribution were found for loading directions *θ* = 0 − 30*°*, *ϕ* = 135*°* and *θ* = 30*°*, *ϕ* = 0 − 355*°* (Figs. 3 & 4). The change in the distributions for both sets of loading directions were similar: the SED was sensitive to both *θ* and *ϕ* in their considered ranges (*θ* = 0 − 30*°* and *ϕ* = 0 − 355*°*). Again, this pattern was consistent across groups and time points.

**Fig. 5:**
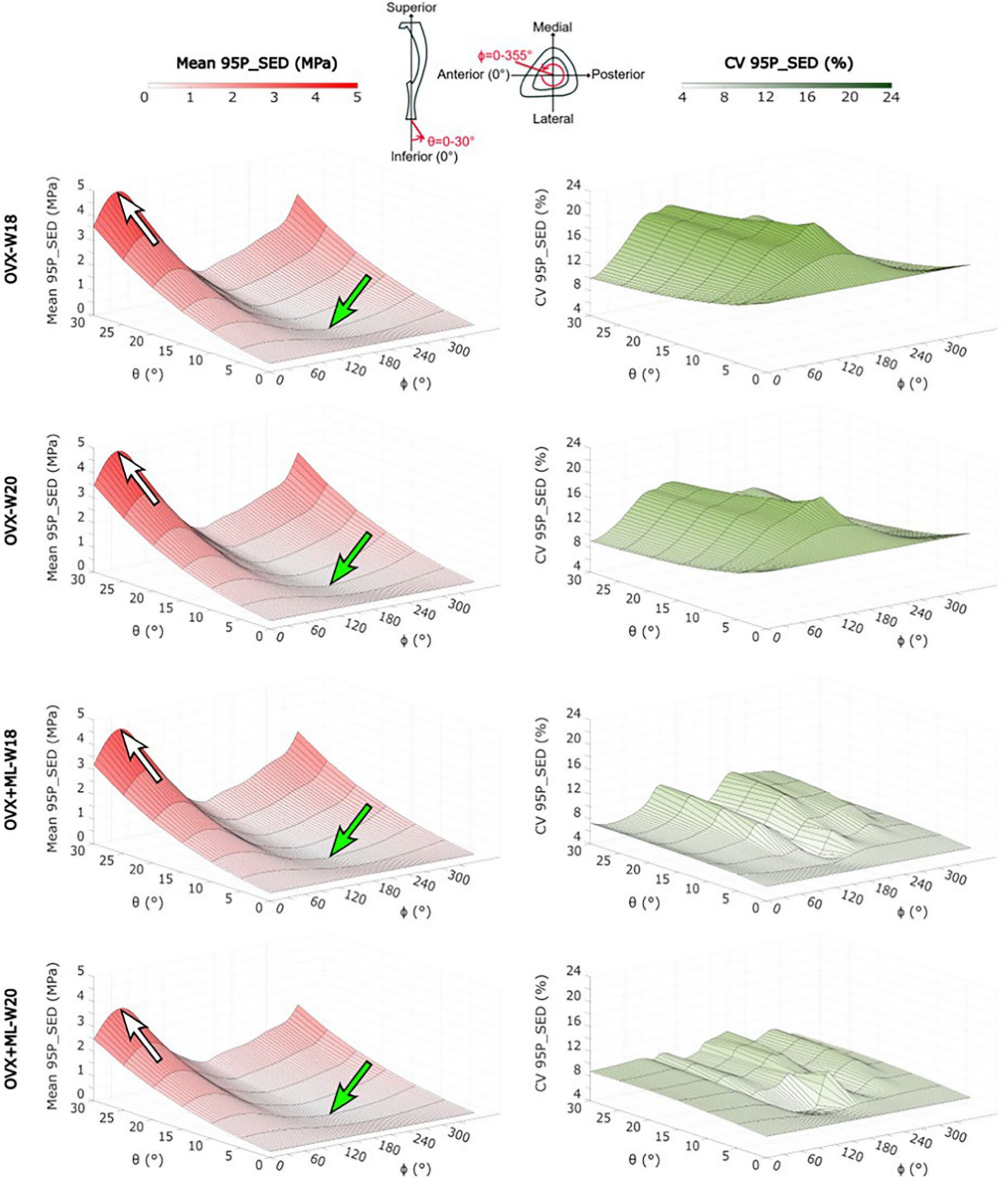
The minimum (green arrow) and maximum (white arrow) mean value of the median of the 95th-100th percentiles of the SED (95P_SED) were found at the same loading direction across groups and time points. OVX, ovariectomy; OVX + ML, ovariectomy and mechanical loading; W18, week 18; W20, week 20

The minimum and maximum values of the 95P_SED were calculated, and their associated loading directions are reported in Table 1. Similar loading directions led to the minimum and maximum values of 95P_SED across the groups. The minimum 95P_SED (Fig. 5, green arrows) was found at *θ* = 10*°*, *ϕ* = 205*°* for both the OVX and OVX + ML groups at both time points. The maximum 95P_SED (Fig. 5, white arrows) was found at *θ* = 30*°*, *ϕ* = 40*°* for the OVX group at both time points and at *θ* = 30*°*, *ϕ* = 45*°* for the OVX + ML group at both time points. The 95P_SED ranged from 0.08–4.79 MPa for the OVX-W18 group, 0.08–4.70 MPa for the OVX-W20 group, 0.07–4.44 MPa for the OVX + ML-W18 group, and 0.06–3.52 MPa for the OVX + ML-W20 group. All the groups and time points showed similar patterns. However, the OVX + ML-W20 exhibited a lower maximum 95P_SED compared to the other groups and time points (Table 1 for specific p-values). The CV ranged from 10.0% to 21.8% for the OVX-W18 group, from 9.0% to 18.8% for the OVX-W20 group, from 4.4% to 11.3% for the OVX + ML-W18 group, and from 5.4% to 11.2% for the OVX + ML-W20 group (Table 2). When comparing longitudinally, the loading direction associated with the minimum CV was within a range of 15*°* for *ϕ* for the OVX and OVX + ML groups, and the loading direction associated with the maximum CV was within a range of 10*°* for *ϕ* for the OVX group. However, the loading direction associated with the maximum CV differed in both *θ* and* ϕ* for the OVX + ML group (*θ* differed by 20*°* and *ϕ* differed by 90*°*).

**Table 1:**
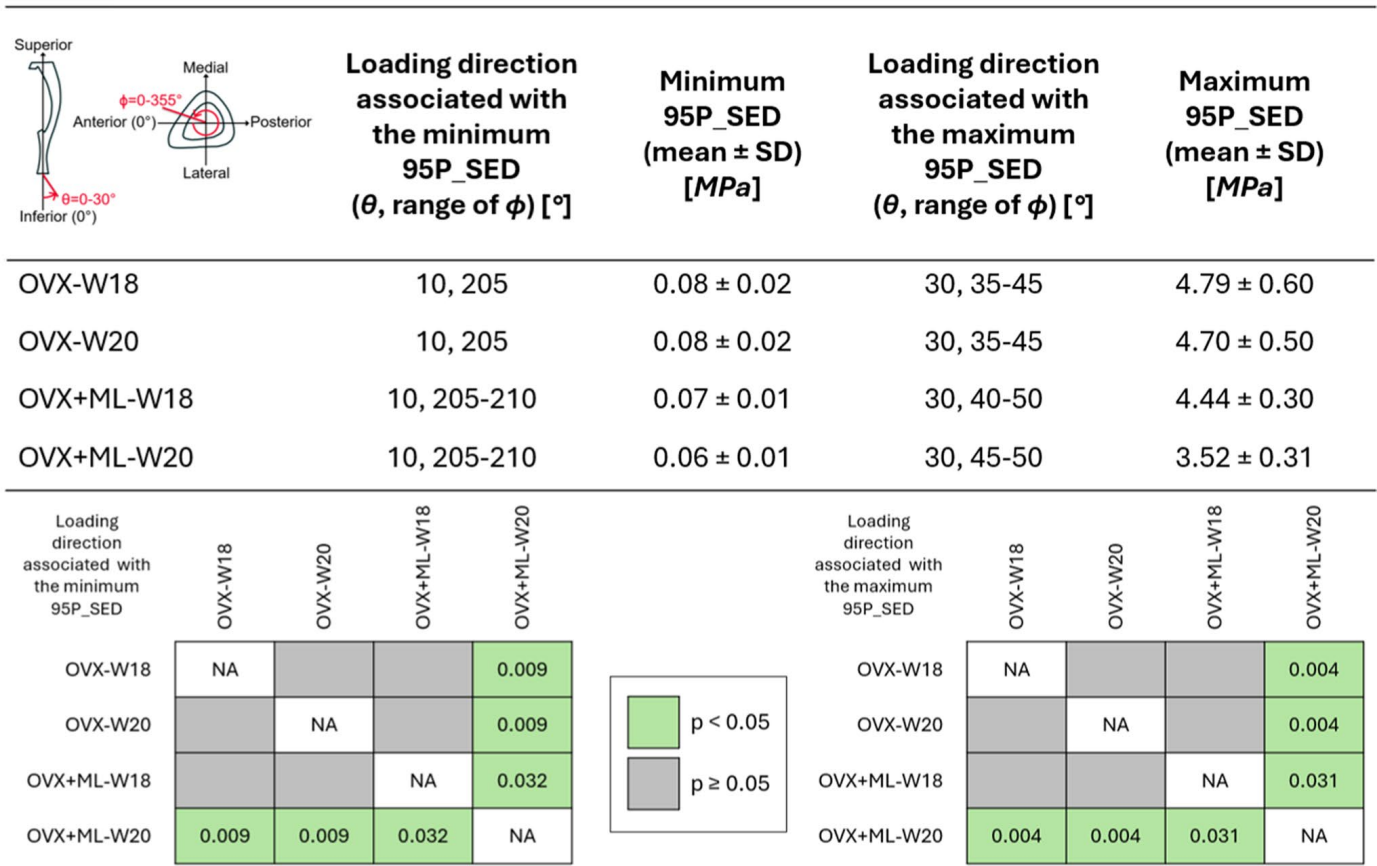
For both groups and time points, the loading directions associated with the minimum and maximum median of the 95th-100th percentiles of the SED (95P_SED) were found at similar loading directions. The *p*-values corresponding to significant differences in the minimum and maximum 95P_SED between the groups and time points can be seen in the bottom grids. As at W18 both groups were untreated, the differences are associated mainly with different animals in the two groups (the differences between OVX-W18 and OVX + ML-W18 for the maximum and minimum 95P_SED are both not significant (*p* ≥ 0.05). OVX, ovariectomy; OVX + ML, ovariectomy and mechanical loading; W18, week 18; W20, week 20

**Table 2:** The minimum coefficient of variation (CV) of the median of the 95th-100th percentiles of the SED (95P_SED) were in a similar range of loading directions for both time points, for each group. Similar trends were found for the maximum CV. For both groups and time points: the loading directions associated with the minimum and maximum CV of the 95P_SED between mice, and the corresponding CV. OVX, ovariectomy; OVX + ML, ovariectomy and mechanical loading; W18, week 18; W20, week 20

	Loading direction associated with the minimum CV(θ, ϕ) [°]	Minimum CV [%]	Loading direction associated with the maximum CV(θ, ϕ) [°]	Maximum CV [%]
OVX-W18	30, 0	10.00	10, 185	21.78
OVX-W20	30, 0	9.03	10, 195	18.79
OVX+ML-W18	10, 190	4.44	30, 120	11.34
OVX+ML-W20	10, 175	5.41	10, 210	11.18

**Fig. 6:**
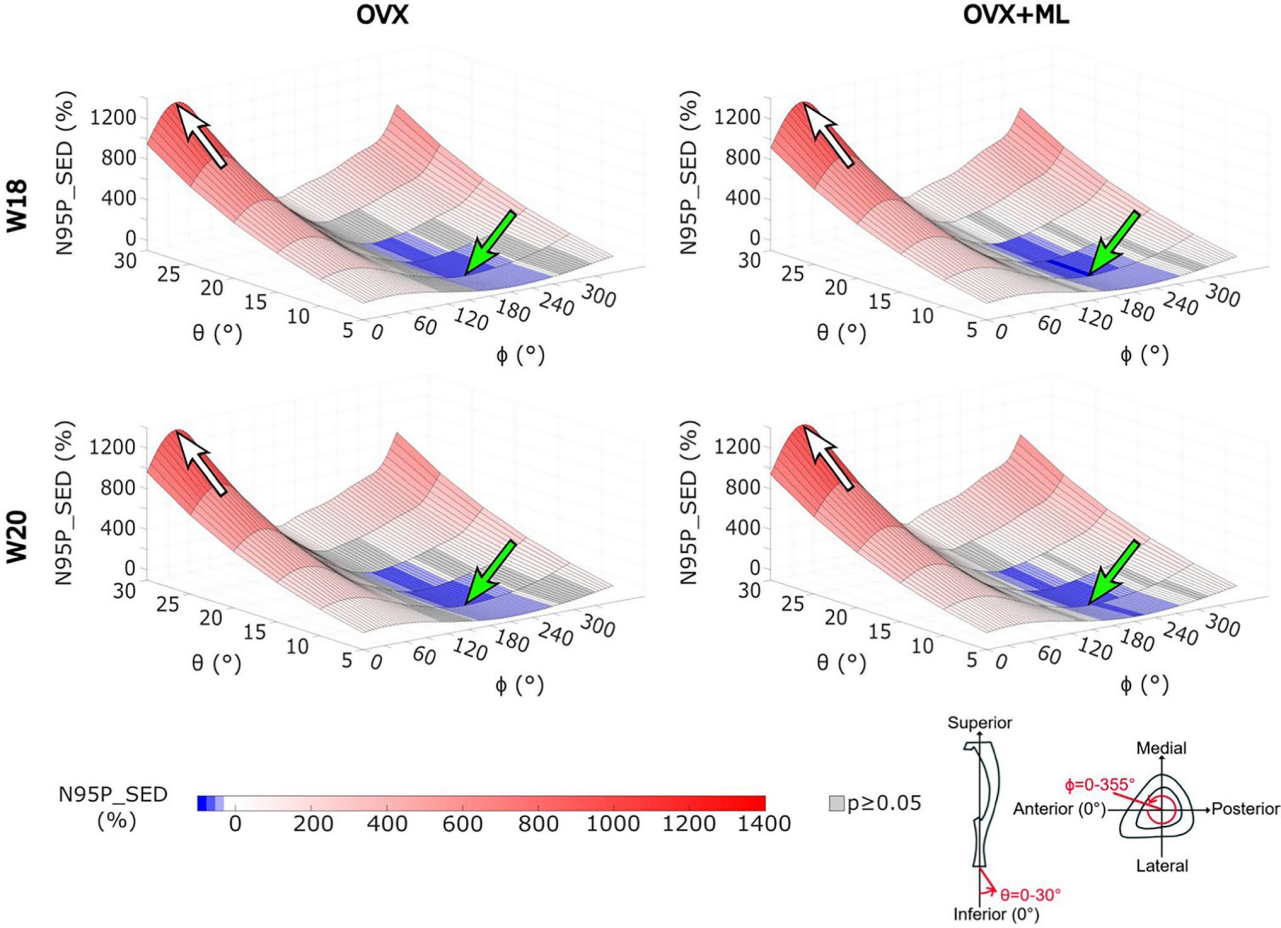
The mean values of the median of the 95th–100th percentiles of the SED ranged from half (highlighted by the green arrow) to fifteen times (highlighted by the white arrow) of that of the nominal axial loading direction. The values in grey show the loading directions associated with a 95P_SED which are not significantly different to the 95P_SED obtained for the nominal axial loading direction (Wilcoxon test, *p* < 0.05). OVX, ovariectomy; OVX + ML, ovariectomy and mechanical loading; W18, week 18; W20, week 20

The N95P_SED ranged from half to fifteen times that of the nominal axial case (Fig. 6). Similar trends were found to the 95P_SED: the maximum N95P_SED was found for *θ* = 10*°*, *ϕ* = 205*°* for all groups and time points. The minimum N95P_SED was found for the loading direction *θ* = 30*°*, *ϕ* = 40*°* for the OVX group and at *θ* = 30*°*, *ϕ* = 45*°* for the OVX + ML group. For all groups and time points, loading directions less than or equal to *θ* = 15*°* and *ϕ* = 175 − 245*°*, a reduction in the N95P_SED was found compared to the nominal axial case.

**Fig. 7:**
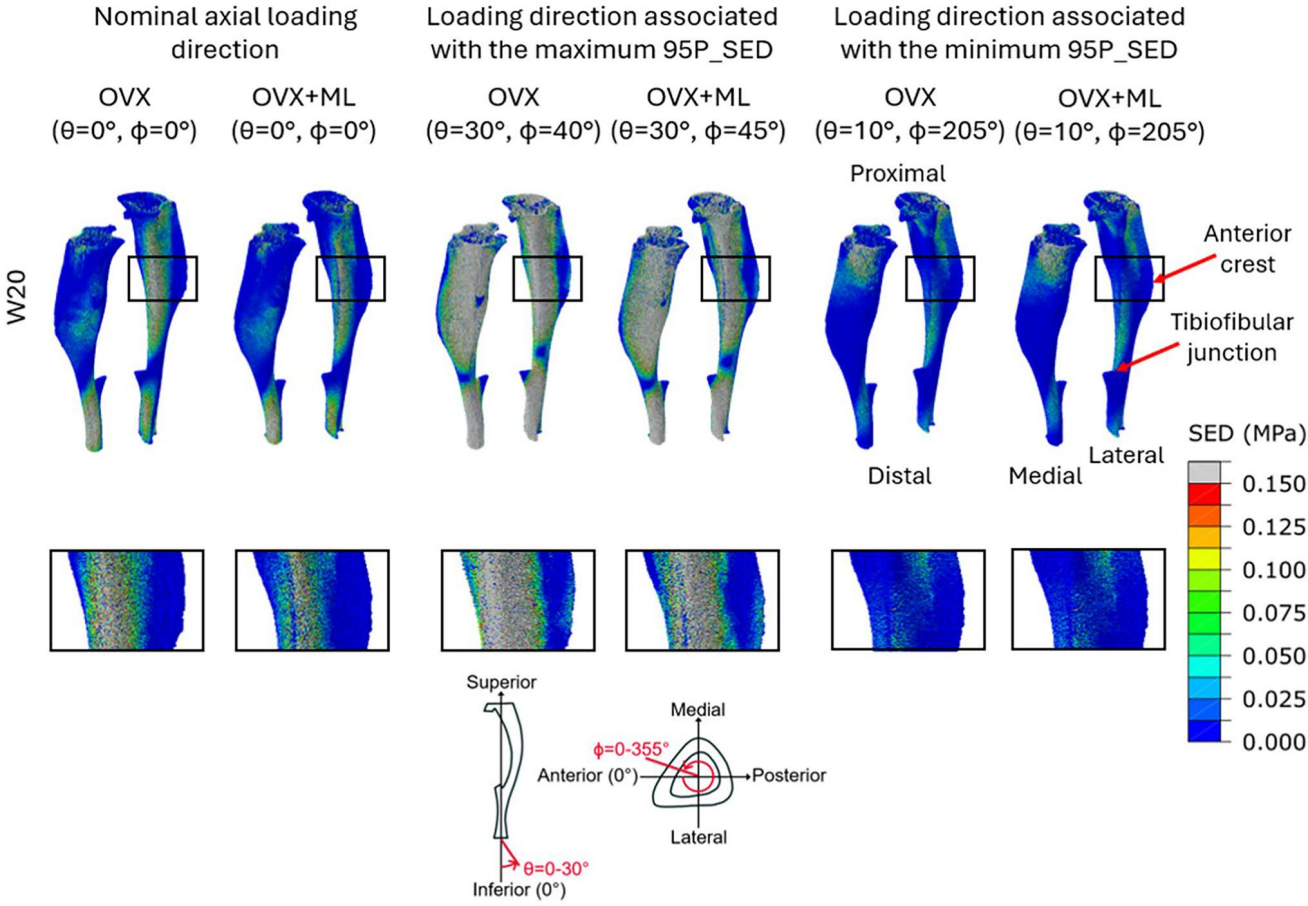
Large difference in the strain energy density (SED) distributions across the different loading directions. SED distributions of two representative tibiae (OVX–group, mouse 2; OVX + ML group, mouse 5) obtained using a load of magnitude 12 N at W20. Left: Load applied along the nominal axial loading direction (θ = 0°, ϕ = 0°). Centre: Load applied along the loading directions associated with the maximum median of the 95th-100th percentiles of the SED (95P_SED) (θ = 30°, ϕ = 40 − 45°). Right: Load applied along the loading direction associated with the minimum 95P_SED (θ = 10°, ϕ = 205°). Bottom row: Close-up view of the area surrounding the anterior crest. OVX, ovariectomy; OVX + ML, ovariectomy and mechanical loading; W20, week 20

**Fig. 8:**
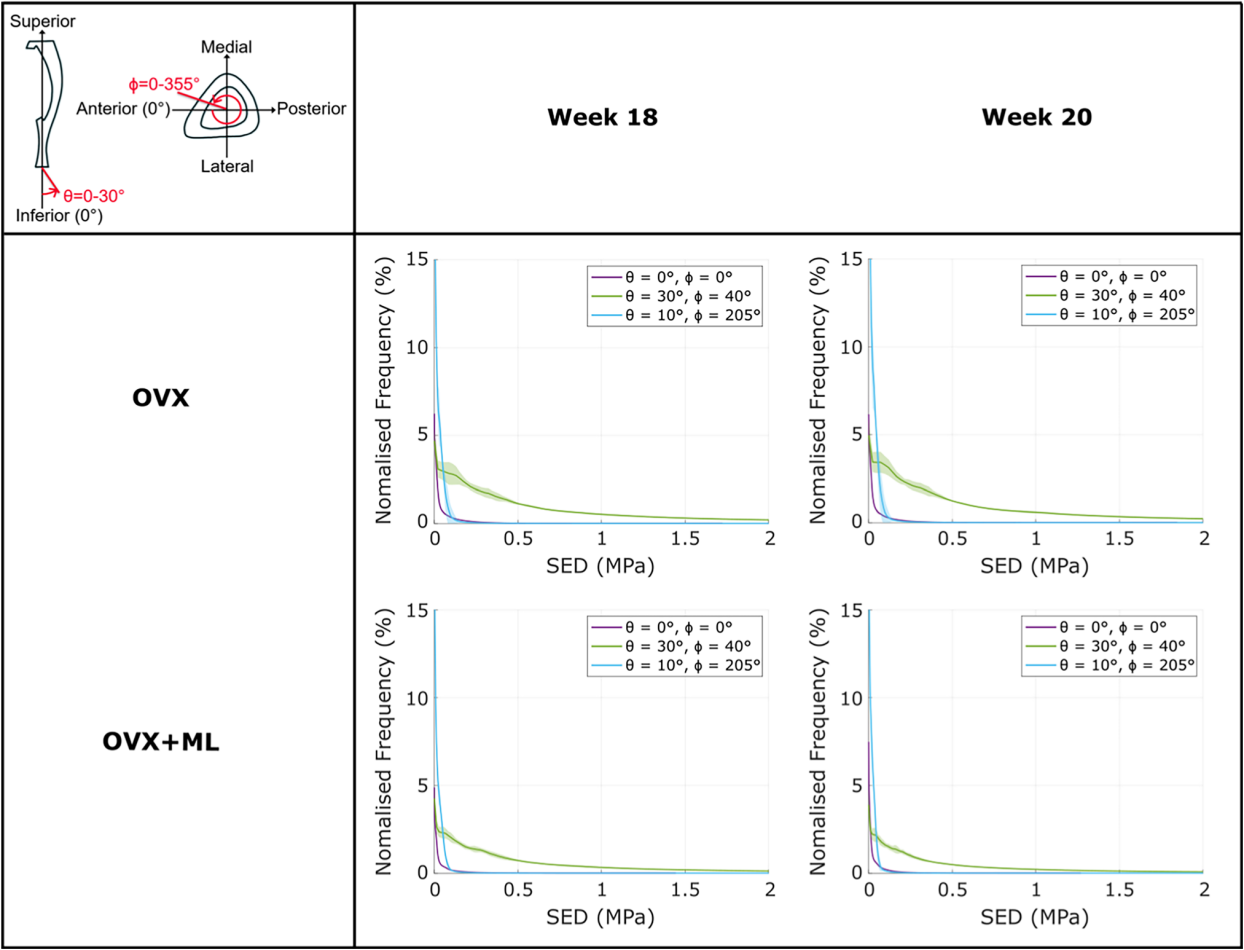
The loading direction associated with the minimum median of the 95th-100th percentiles of the SED (95P_SED) had a higher frequency of strain energy density (SED) values close to 0 MPa compared to the nominal axial case. The frequency plots for the SED (mean ± standard deviation) between 0 and 2 MPa, for the loading directions associated with the minimum 95P_SED (θ = 10°, ϕ = 205°), the maximum 95P_SED (θ = 30°, ϕ = 40 − 45°), and the nominal axial case (θ = 0°, ϕ = 0°), for both groups at both time points. OVX, ovariectomy; OVX + ML, ovariectomy and mechanical loading; W18, week 18; W20, week 20

Typical spatial distributions of the SED obtained for the nominal axial loading direction (*θ* = 0*°*, *ϕ* = 0*°*) and for the loading direction associated with the maximum or the minimum 95P_SED values are reported in Fig. 7. Large differences were observed in the SED distributions across the different loading directions. The loading direction associated with the minimum 95P_SED (*θ* = 10*°*, *ϕ* = 205*°*) had a higher frequency of values close to 0 MPa compared to the nominal axial case (Figs. 7 & 8). Similarly, the loading directions associated with the maximum 95P_SED (*θ* = 30*°*, *ϕ* = 40 − 45*°*) had higher SED values compared to the nominal axial case (Figs. 7 & 8). For the nominal axial loading direction, high values of the SED were found mainly in the distal portion of the tibia and across the posterior side of the tibia. Conversely, in the anterior crest of the mouse tibia, lower SED values were found. However, the SED in the anterior crest was higher for the case with maximum 95P_SED, compared to the nominal axial case. High SED values were found close to the distal tibiofibular junction, in the elements situated more proximally, especially in the posterior portion of the bone, across all groups and time points. As expected, considering that the interventions started at week 19 of age, small differences were found for models at week 18 between the two groups (both groups untreated), and larger differences induced by the mechanical loading were observed at week 20.

**Fig. 9:**
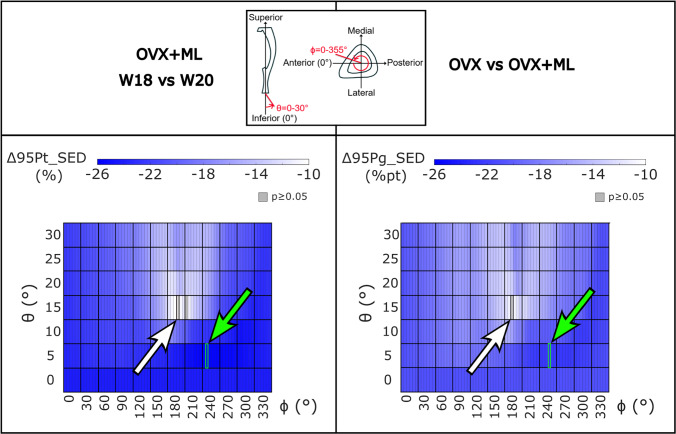
The percentage difference in the median of the 95th-100th percentiles of the SED (∆95Pt_SED) for the OVX + ML group across time points were statistically significant for all loading directions except one, and the percentage point differences between groups across time points (∆95Pg_SED) were significantly different for every loading direction. Left: Heatmaps of the ∆95Pt_SED between time points (W18 vs W20) for the OVX + ML group (Wilcoxon test, p < 0.05). The value in grey shows the loading directions associated with a non-significant difference between time points (θ = 15°, ϕ = 210°) (i.e., *p* ≥ 0.05). Right: Heatmaps of difference in ∆95Pt_SED for the OVX and OVX + ML groups (∆95Pg_SED; percentage point) for all loading directions. All differences are statistically significant (Mann–Whitney U test, *p* < 0.05). The black square and white arrow (pointing upwards) highlight the loading direction for which the smallest difference was found, and the green square and arrow (pointing downwards) highlight the loading direction for which the largest difference was found. OVX, ovariectomy; OVX + ML, ovariectomy and mechanical loading; W18, week 18; W20, week 20

For the OVX group, the 95P_SED between weeks 18 and 20 (∆95Pt_SED) were not significantly different for any loading direction (maximum absolute difference 4.2%, *p* ≥ 0*.*05; details in the Supplementary Figure S1). For the OVX + ML group, the 95P_SED decreased significantly between weeks 18 and 20 for all except one loading direction. The range of the ∆95Pt_SED for the OVX + ML group was between -25.6% (*θ* = 5*°*, *ϕ* = 245*°*) and -9.8% (*θ* = 15*°*, *ϕ* = 195*°*) (Fig. 9). Percentage differences between ∆95Pg_SED for the OVX and the OVX + ML groups were significant for every loading direction (*p* < 0*.*030), with ∆95Pg_SED ranging between -22.5% (*θ* = 5*°*, *ϕ* = 255*°*) and -10.8% (*θ* = 15*°*, *ϕ* = 190*°*) (Fig. 9).

## Discussion

The purpose of this study was to quantify the effect of the loading direction on the SED distribution within the mouse tibia after applying external mechanical loading. Using validated micro-CT based micro-FE models, the SED distributions across the tibiae were calculated for several loading directions.

The frequency plots showed that the SED experienced by the bone is sensitive to changes in the loading direction, both in the inferior-superior direction and in the anterior–posterior and medio-lateral directions. As *θ* increased, larger transverse loads were applied to the model, resulting in an increase of high SED values compared to the nominal axial case. The SED distributions across the tibiae for the nominal axial case were in line with those from a previous study, which applied the same load magnitude (Cheong et al. 2021a). Additionally, the high SED values, found proximally to the distal tibiofibular junction, in the posterior portion of the bone for all groups and time points, are consistent with locations of bone formation reported experimentally after the application of external mechanical loading (Roberts et al. 2020; Sugiyama et al. 2012) and predicted by a mechanoregulation model (Cheong et al. 2020b). As expected, the values of the maximum 95P_SED for the different loading directions showed that the bone experiences high SED values when loaded in the direction occurring at *θ* equal to 30*°* and *ϕ* equal to 35 − 50*°*. This variation in SED distribution could help explain some of the differences observed in the current literature regarding the location of bone formation after applying external mechanical loading by using the in vivo tibial loading model. For example, Pereira et al. (2015) observed that after applying a 13*N* external mechanical load to the tibia, bone formation occurred on the medial surface of the tibia and at the interosseous crest (the prominent ridge along the lateral side of the tibia). Whilst Carriero et al. (2018) observed that after applying a 12*N* external mechanical load, the majority of bone formation happened on the lateral side, and on a moderate amount on the medial side of the tibia. Additionally, the anterior crest and the surrounding area undergo an increase in SED when loaded in the direction associated with the maximum 95P_SED. This spatial location is where the largest changes in bone mineral density (Roberts et al. 2020) and periosteal formation (Cheong et al. 2020b) have been observed after applying external mechanical loading to the bone.

Furthermore, higher strains are associated with increased woven bone formation (Sugiyama et al. 2012). Therefore, it remains to be investigated if increasing the transverse loads in the tibia loading model would lead to woven or lamellar bone formation. Moreover, as the bone experiences higher SED values, it predisposes it to fracture when loaded in this direction, compared to the nominal axial case. This is in line with a recent study performed within the group on the same dataset, which showed that the failure load of the mouse tibia was lowest for a load in the direction occurring at *θ* equal to 30*°* and *ϕ* equal to 30 − 50*°* (Farage-O’Reilly et al. 2024). The loading directions corresponding to the maximum SED values fall within this range. Therefore, although loading directions associated with *θ* equal to 30*°* and *ϕ* equal to 30 − 50*°* may induce more bone formation, a good compromise between the loading direction and the load magnitude should be found to avoid increasing the risk of inducing micro-damage or even fractures during the experiment.

Conversely, the loading directions associated with the minimum 95P_SED occur when *θ* equal to 10*°* and *ϕ* equal to 205 − 210*°*. These results are in line with a recent study, which reported the same loading direction to be associated with the highest failure load (Farage-O’Reilly et al. 2024). Due to this reduction in high SED values, it is plausible that less mechanoregulated bone formation would occur if loaded in this direction compared to the nominal axial case. This result suggests that bone is optimised for that loading direction. Additionally, given that the fibula’s typical orientation is at a *ϕ* angle ranging from 170*°* to 220*°*, the fibula may share some of the load and reduce these high SED values if the tibia is loaded along these directions. The standard deviations for each loading direction between 0 MPa and 2 MPa are consistently low, highlighting the reproducibility of the results and suggesting a good registration between tibiae. Poor registration would likely result in greater variation in results due to misalignment of the loading directions. Furthermore, the loading direction associated with the minimum and maximum 95P_SED are found within *ϕ* = 205–210*°* and *ϕ* = 35–50*°* across groups and time points, respectively. This small range in the loading direction suggests that the external mechanical loading had minimal impact on the loading direction associated with the maximum 95P_SED. However, the coefficient of variation for the 95P_SED ranges from 4.4% to 21.8% across the groups and time points. This suggests that the larger SED values experienced by each mouse across the loading directions varied. This could be due to differences in geometry and microstructure among tibiae and between groups, as reported by Moraiti et al. (2024).

The local distributions of SED in the mouse tibia have been shown to be very sensitive to the loading direction, with some loading directions resulting in a 95P_SED ranging from half to fifteen times that of the nominal axial case. A previous study, which used a minimum principal strain failure criterion, showed that the failure load ranged from half to double that of the nominal axial case, for the same range of loading directions (Farage-O’Reilly et al. 2024). These results expand on those of a previous study, which showed that a small load along the anterior–posterior direction on top of an axial compressive load affects the local deformation and SED (Cheong et al. 2021a). Similar concerns regarding loading direction have been identified in previous studies, including a repositioning investigation for the in vivo tibial loading model (Giorgi and Dall’Ara 2018) and a misalignment study for the rodent tail loading model (Goff et al. 2014). It has been suggested that the loading direction could be controlled through the use of a tri-axial load cell during the loading procedure, or through the use of mouse-specific 3D printed loading caps (Farage-O’Reilly et al. 2024; Main et al. 2020; Meakin et al. 2014). Controlling well the variability of the loading direction could improve predictions of bone adaptation when using mechanoregulation models, as it would limit the propagation of errors in the strain distributions being carried forward through the model pipeline (Cheong et al. 2020b, 2021b; Pereira et al. 2015). Without more accurate experimental techniques to accurately control the loading direction assigned in the in vivo tibia model, the computational models to predict bone adaptation may benefit from incorporating the uncertainties in the loading direction when predicting the stress/strain distribution and the mechanical stimulus. When comparing the 95P_SED between time points, the OVX group showed no statistically significant differences for all loading directions. This is corroborated by Farage-O’Reilly et al. (2024), where it was shown that the failure load for the OVX group between weeks 18 and 20 were not statistically different for all the loading directions (an extension on studies by Roberts et al. (2019), (2024)), who showed no change in the failure load between weeks 18 and 20 for the assumed nominal axial case for the OVX group). In contrast, the OVX + ML group showed a statistically significant difference in the 95P_SED between the time points for all loading directions, except one (a decrease between 9.8% and 25.6%). Furthermore, a statistically significant decrease in ∆95Pg_SED was found for all loading directions (between − 10.8% and − 22.5%). These results suggest that the external mechanical loading changed the morphometric and densitometric properties of the bone, as previously reported by Roberts et al. (2020). In fact, in that study, after four weeks of treatment, the proximal trabecular bone volume fraction and the diaphyseal cortical thickness were found to increase by 89% and 27%, respectively. Interestingly, the direction of the optimal load exhibited minimal variation (within *ϕ* = 5*°*) across groups and time points (Fig. 5). This suggests a relatively uniform increase in the 95P_SED throughout the tibia, regardless of loading direction during mechanical loading.

This study is limited by the removal of the fibula from the model. An increase in *θ* and/or *ϕ*, increases the transverse load applied to the tibia. These loads would in part be shared with the fibula (Cheong et al. 2021a; Prasad et al. 2010). Therefore, the inclusion of the fibula in the model is expected to result in lower SED values for loading directions which have larger transverse load components, as suggested by Yang et al. (2014). However, further investigation is needed to quantify the extent to which the fibula contributes to mitigating high tibial SED values, especially for transverse loads. Moreover, the tibiofibular joint properties are currently unknown, which would need the introduction of additional assumptions to the modelling pipeline to include the fibula. Similarly, the growth plate has been removed from the model, as including this would introduce additional assumptions about the unknown growth plate material properties. Instead, it was assumed that the load is uniformly applied to the proximal slice of the model. However, it has recently been found that the strains in the tibia are highly sensitive to the load location (a study done using micro-FE models and strain gauges, to inversely identify the location of the load applied) (Pickering et al. 2021). Therefore, it would be of value to model a range of loading applications in future iterations of the model. This could allow for a more accurate assessment of load distribution and its influence on the SED experienced by the bone. Furthermore, it is acknowledged that the exact in vivo loading conditions on the tibia are difficult to know and that complexities such as alignment errors and distribution of the load through the articular surface were not explicitly modelled. While in this study we have assessed how the SED in the tibia is affected only by the loading direction, it is implicitly influenced by the above-mentioned complexities. Other factors beyond the loading direction may also affect bone adaptation. For example, the influence of the soft tissues, which may alter load transmission and distribution in vivo, the effect of muscle forces and of local joint inflammation will be explored in future studies.

This study has revealed the highly sensitive nature of the SED distribution within the mouse tibia to the loading direction. These findings have significant implications for both experimental design and computational modelling of bone adaptation. Accounting for the observed variations in the SED can reduce uncertainties in experimental measurements and enhance the predictive power of multiscale models, potentially leading to more accurate simulations of mechanoregulated bone adaptation. Future research should extend these findings to examine if these observed changes in SED distribution result in predictive changes in bone adaptation.

## Supplementary Information

Below is the link to the electronic supplementary material.

Supplementary file1 (PDF 238 KB)

## Data Availability

Example of the raw data and dataset used to generate the figures can be found in the following Figshare link (10.15131/shef.data.29994211). If the reader is interested in the raw data, they can contact the corresponding author.
