# Supplementary material for: Quantifying the local strain energy density distribution in the mouse tibia: the critical role of the loading direction
Source: Biomech Model Mechanobiol. 2025 Sep 3;24(6):2153–69. doi: 10.1007/s10237-025-02011-z (PMC12618303; doi:10.1007/s10237-025-02011-z)
Supplement: Supplementary file 1 — Supplementary file1 (PDF 238 KB) [file 10237_2025_2011_MOESM1_ESM.pdf]

Quantifying the local strain energy density distribution in the mouse tibia: the critical role of the loading direction

Saira Mary Farage-O'Reilly[1,2,3], Vee San Cheong[1,4], Peter Pivonka[5,6], Visakan Kadirkamanathan[1,7], Enrico Dall'Ara[1,2,3*]

[1*]Insigneo Institute, University of Sheffield, Sheffield, United Kingdom.
[2]Healthy Lifespan Institute, University of Sheffield, Sheffield, United Kingdom.
[3]Division of Clinical Medicine, University of Sheffield, Sheffield, United Kingdom.
[4]School of Mechanical, Aerospace and Civil Engineering, University of Sheffield, Sheffield, United Kingdom.
[5]School of Mechanical, Medical and Process Engineering, Queensland University of Technology, Brisbane, QLD, Australia.
[6]Centre for Biomedical Technologies, Queensland University of Technology, Brisbane, QLD, Australia.
[7]School of Electrical and Electronic Engineering, University of Sheffield, Sheffield, United Kingdom.

Corresponding Author:
E-mail(s): e.dallara@sheffield.ac.uk

Biomechanics and Modeling in Mechanobiology

Supplementary Material

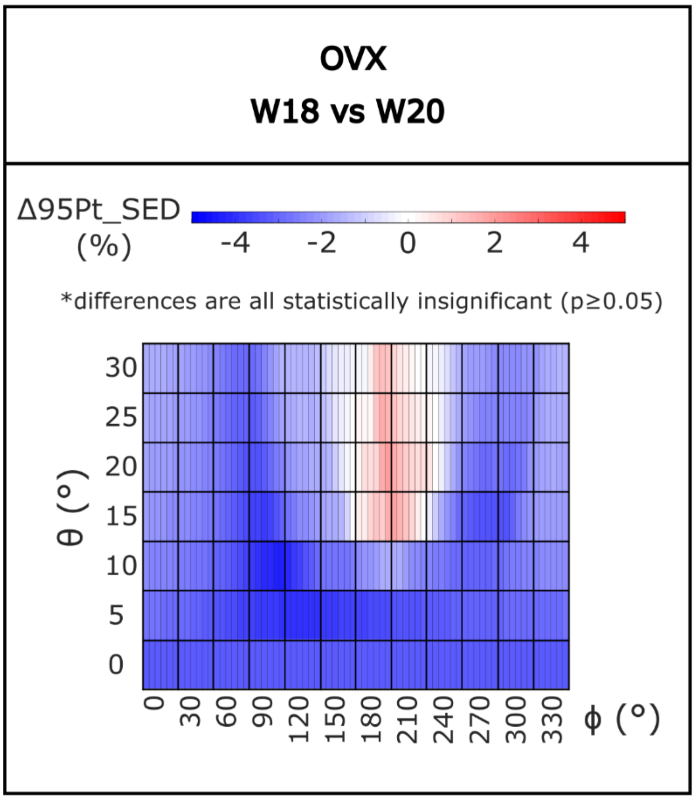

Fig. S1 *Heatmap of the percentage difference in the mean values of the median of the 95th-100th percentiles of the SED (Δ95Pt_SED) between time points (W18 vs W20) for the OVX group, calculated across the region of interest comprising of 10-90% of the cropped tibial length, for all loading directions (θ in range $0 - 30°$, ϕ in range $0 - 355°$). Differences are all statistically insignificant (Wilcoxon test, $p \geq 0.05$). OVX, ovariectomy; W18, week 18; W20, week 20*
